# Molecular characterization of enterohemorrhagic *Escherichia coli* isolated from diarrhea samples from human, livestock, and ground beef in North Jordan

**DOI:** 10.14202/vetworld.2021.2827-2832

**Published:** 2021-10-30

**Authors:** Yaser H. Tarazi, Saeb N. El-Sukhon, Zuhair Bani Ismail, Amani A. Almestarehieh

**Affiliations:** 1 Department of Basic Medical Veterinary Sciences, Faculty of Veterinary Medicine, Jordan University of Science and Technology, Irbid, Jordan; 2 Department of Veterinary Clinical Sciences, Faculty of Veterinary Medicine, Jordan University of Science and Technology, Irbid, Jordan.

**Keywords:** animal health, Enterobacteriaceae, foodborne pathogens, public health

## Abstract

**Background and Aim:**

Enterohemorrhagic *Escherichia coli* (EHEC) is an important foodborne pathogen with worldwide distribution. Data regarding its presence, distribution, virulence, and antimicrobial susceptibility among various animal species and humans in Jordan are lacking. Therefore, the objectives of this study were to isolate and characterize EHEC from human and animal diarrhea fecal samples and ground beef samples.

**Materials and Methods:**

A total of 100 and 270 diarrhea fecal samples from humans and animals, respectively, were collected. In addition, 40 ground beef meat samples were collected from retail markets. EHEC was positively identified by detecting Shiga toxins (*stx1* and *stx2*) genes using multiplex polymerase chain reaction (PCR). Antimicrobial susceptibility patterns were determined using the disk diffusion test. Beta-lactamase production was detected using the double disk diffusion test and the extended-spectrum beta-lactamases (ESBLs) were identified by detection of *bla_TEM_*, *bla_SHV_*, and *OXA-1* genes using multiplex PCR. Pulsed-field gel electrophoresis (PFGE) was used to investigate the relatedness of EHEC isolates from different sources.

**Results:**

Out of 410 samples, 194 *E. coli* isolates were positively identified, of which 57 isolates (29%) were classified as EHEC. Thirty-five (61%) of EHEC isolates were serotyped as O157 (19: O157:H7 and 16: O157:NM). The *stx1* gene was detected only among the sheep and goats isolates at a rate of 7.6% and 5.2%, respectively, while the *stx2* gene was detected in only one ground beef meat sample. EHEC isolates showed high resistance patterns against amoxicillin, gentamycin, cephalexin, and doxycycline. Twenty-four out of 32 EHEC isolates were determined as ESBL producers, among which 14 isolates expressed the *bla_SHV_*gene and 19 isolates expressed the *bla_TEM_* while four expressed both genes. PFGE analysis revealed two clusters with high similarity (92%) originated from ground beef meat and cattle fecal samples. No similarities were found between human and animal *E. coli* isolates.

**Conclusion:**

Results of this study indicate widespread ESBL EHEC among humans, animals, and ground beef meat samples. These results represent an important alarm that requires the implementation of appropriate preventative measures by both human and animal health sectors to prevent the transmission of this important foodborne pathogen.

## Introduction

*Escherichia coli* is a Gram-negative bacillus of the family Enterobacteriaceae [1,2]. Although *E. coli* bacteria are normal inhabitants of the gastrointestinal tract of both humans and animals, pathogenic strains are capable of causing important and potentially fatal diseases [1,2].

In humans, enterohemorrhagic *E. coli* (EHEC) is considered important zoonotic bacteria causing severe bloody diarrhea, hemorrhagic colitis, hemolytic-uremic syndrome, and thrombotic thrombocytopenic purpura [1-5]. In cattle and sheep, several pathogenic *E. coli* are the cause of neonatal calf diarrhea and sepsis, including enterotoxigenic (ETEC), enteropathogenic (EPEC), shigatoxigenic (STEC), enterohemorrhagic (EHEC), enteroinvasive (EIEC), enteroaggregative (EAEC), and enteroadherent *E. coli* (EAdEC) strains [6-10].

Several outbreaks of foodborne illnesses around the world have been traced back to the consumption of poultry and ground meat, dairy products, and fresh vegetables contaminated with *E. coli* O157:H7 [1-3]. This extremely pathogenic strain of *E. coli* is characterized by several virulence factors, including biofilm formation and production of potent toxins, including Shiga toxins, intimin, and enterohemolysin [1-11].

In Jordan, there is a paucity of information regarding the presence, distribution, virulence, and antimicrobial susceptibility of EHEC among various animal species and humans. Therefore, the objective of study was to isolate and characterize EHEC from human and animals’ diarrhea fecal samples, poultry litter, and ground beef samples.

## Materials and Methods

### Ethical approval and informed consent

The study was approved by the Institutional Animal Use and Care Committee of Jordan University of Science and Technology. Informed written and signed consents were obtained from farm owners and human patients before sample collection was carried out.

### Study period and location

The study was carried out from April 2014 and June 2015 at the Research Microbiology Laboratory, Faculty of Veterinary Medicine, Jordan University of Science and Technology, Jordan.

### Sample collection

#### Human samples

A total of 100 fecal swab samples were collected from human patients affected with gastroenteritis characterized by diarrhea and abdominal pain. Patients were of various ages ranging from 15 to 75 years who undergone medical evaluation for acute gastroenteritis in two local medical centers located in North Jordan. Sterile swabs (FecalSwab; Copan, USA) were used to collect fecal samples in an aseptic manner by the patient who was instructed to wear disposable gloves during sample collection. Samples were transported to the laboratory within 2-4 h after collection and were cultured immediately.

#### Animal fecal samples

A total of 270 fecal samples were collected from cattle (n=100), sheep (n=35), goats (n=35), and broiler chicken litter (n=100). Samples were collected aseptically using a gloved hand directly from the rectum (cattle, sheep, and goats) and from fresh poultry litter. Samples were obtained from animals suffering from acute gastroenteritis characterized by diarrhea. Animals belonged to several farms located in Northern and Middle regions of Jordan. Samples were placed in sterile plastic fecal containers with screw caps and transported to the laboratory within 2-4 h after collection for immediate culture.

#### Ground meat samples

A total of 40 ground beef meat samples were collected aseptically from local retail markets. Approximately 100 g of ground meat was placed in sterile containers with screw cap and transported to the laboratory within 2-4 h after collection for immediate culture.

### Bacterial culture and identification

A reference strain of EHEC O157:H7 (ATCC 700728) was obtained from Jordan Food and Drug Administration (Amman, Jordan) and was used as a positive control in the study. Bacterial culture was performed according to previously published protocols [3,12]. Briefly, fecal swabs were inoculated into 9 mL of Mueller-Hinton broth (Oxoid, UK) and incubated at 37°C for 24 h. Then, a loopful was streaked on MacConkey agar medium (Oxoid) and on eosin methylene blue (Oxoid) and incubated overnight at 37°C. Colonies of *E. coli* were identified by biochemical reactions using IMViC test (Tulip Diagnostics, India).

### Identification and serotyping of *E. coli* O157:H7

*E. coli* colonies were inoculated on sorbitol MacConkey agar (Oxoid) and incubated for 18-24 h at 37°C. The isolates that showed the inability to ferment sorbitol with colorless or pale color colonies were predicted to be O157:H7. Suspected O157:H7 isolates were further serotyped using microtiter plate agglutination against nine specific O-antisera, including O157, O8, O26, O45, O121, O145, O113, O103, and O111 (Statens Serum Institute, Copenhagen, Denmark).

### DNA extraction

DNA was extracted using cell lysis method [13]. Briefly, 40 μL of SCLB (1 mL of 1× concentration of Tris-Borate-EDTA [TBE] buffer [Bio Basic, Canada] plus 10 μL of proteinase K [5 mg/mL]) were added to individual sterile polymerase chain reaction (PCR) tubes. A single colony of *E. coli* from MHA was added as DNA template. Amplification was carried out using in BioRad MyCycler™ (Bio-Rad, California, USA) at 80°C for 10 min followed by one cycle at 55°C for 10 min. Then, 80 μL of ddH_2_O was added, centrifuged at 7000 rpm for 2 min, and finally, the extracted templates were stored at 4°C until used.

### Molecular confirmation of *E. coli*

*E. coli* was confirmed by PCR using universal *E. coli* Eco-1 and Eco-2 primers (Table-1) [12-14]. The reference strain ATCC 22925 and normal saline were used as positive and negative control, respectively. The PCR reaction mixture was prepared from 2 μL of DNA template, 2 μL of MgCl_2_ (25 Mm MgCl_2_, KAPA), 10 μL of 2× PCR master mix solution (Intron, South Korea), 1 μL (10 pmoles) of each primer, and 4 μL of nuclease-free water (IDT, Coralville, Iowa, USA) in a final volume of 20 μL. Amplification was carried out using BioRad MyCycler™ (Bio-Rad), at 94°C for 3 min, followed by 35 cycles of 94°C for 30 s, annealing at 62°C for 1 min, and extension at 72°C for 30 s, with a final extension at 72°C for 7 min. The products were subjected to electrophoresis on 1% agarose gels (CSL-AG 100) containing ethidium bromide (10 mg/mL solution). Molecular ladder with 100 bp of size was used as a marker (GeneDireX, Taiwan). Agarose gel electrophoresis was performed in 1× TBE buffer at 100 V for 35 min and finally visualized under UV light (Alpha Innotech, USA).

**Table-1:** Primer sequence and product size of target genes used to detect *Escherichia coli* and its virulence factors.

Primer sets	Target gene	Primer sequence 5’- 3’	Product size (bp)
Eco	*malB* promoter	F: GACCTCGGTTTAGTTCACAGA R: CACACGCTGACGCTGACCA	585
Rfb	*O157*	F: GTGTCCATTTATACGGACATCCATG	292
		R: CCTATAACGTCATGCCAATATTGCC	
sxt1	*Stx1*	F: TGTAACTGGAAAGGTGGAGTATAC	210
		R: GCTATTCTGAGTCAACGAAAAATAAC	
sxt2	*Stx2*	F: GTTTTTCTTCGGTATCCTATTCCG	484
		R: GATGCATCTCTGGTCATTGTATTAC	
FliCh7	*H7*	F: GCGCTGTCGAGTTCTATCGAGC	625
		R: CAACGGTGACTTATCGCCATTCC	
Multi TSO-T	*bla_TEM_*	F: CATTTCCGTGTCGCCCTTATTC R: CGTTCATCCATAGTTGCCTGAC	800
Multi TSO-S	*bla_SHV_*	F: AGCCGCTTGAGCAAATTAAAC R: ATCCCGCAGATAAATCACCAC	713
Multi TSO-O	*OXA-1*	F: GGCACCAGATTCAACTTTCAAG R: GACCCCAAGTTTCCTGTAAGTG	564

### Detection of stx1, stx2, and O157:H7 genes

Shiga toxins 1 and 2, *O157* (RfbE), and *H7* (Flich7) target genes were detected in isolated *E. coli* strains using multiplex PCR. The PCR reaction was prepared in 20 μL final volume which contained 8.2 μL of nuclease-free water, 5 μL of the DNA template, 4 μL of ready 5× Hot FIREPol® Blend Master Mix Ready to load [Hot FIREPol® DNA Polymerase, proofreading enzyme, 5× Blend Master Mix Buffer, 12.5 mM Mgcl_2_, 2 mM dNTPs (Solis Biodyne, Estonia)], and selected primers (Table-1) to a final concentration of 10 μM [7,15]. The thermocycling conditions were set at initial denaturation of 95°C for 15 min, followed by 25 cycles of 94°C for 30 s, 65°C for 90 s, 72°C for 90 s, and final extension at 72°C for 7 min. Amplified samples were evaluated by 1.2% agarose gel electrophoresis in 1× of TBE buffer, ethidium bromide (0.5 μg/mL) staining and visualized under UV illumination [16].

### Antimicrobial susceptibility test

Antimicrobial susceptibility test was performed using the Kirby–Bauer disk diffusion method on MHA according to the recommendations of the Clinical and Laboratory Standard Institute guidelines [17]. Sixteen different antimicrobial agents were used (Bioanalyse, Turkey) including doxycycline (DO 30 μg), amoxicillin (AX 25 μg), gentamicin (CN 10 μg), (ciprofloxacin 5 μg), (levofloxacin 5 μg), (florfenicol 30 μg), (cefepime 30 μg), (aztreonam 30 μg), (imipenem 10 μg), (ceftriaxone 30 μg), (cefoxitin 30 μg), cephalexin (CL 30 μg), (cefuroxime 30 μg), (cefotaxime 30 μg), ceftazidime (CAZ 30 μg), and (trimethoprim/sulfamethoxazole 25 μg). Antimicrobial susceptibility was reported as a measure of the diameter of the inhibition zone in millimeters after incubation at 37°C for 24 h.

### Double-disk synergy test (DDST)

The DDST was used to detect extended-spectrum beta-lactamase (ESBL) genes among EHEC isolates [17]. Briefly, isolates were inoculated on MHA plates. Then, an AX/clavulanic acid disk was placed in the center of the plate. Then, CAZ and ceftizoxime disks were placed at 25 mm from the center and away from the AX/clavulanic acid disk. The plates were then incubated at 37°C for 24 h. An increase of >5 mm of the inhibition zone was recorded as a positive result. A reference strain of *E. coli* (ATCC 700728) was used for comparison as a positive control.

### Detection of bla_TEM_, bla_SHV_, and OXA-1 genes

ESBL-positive isolates were used to detect the chromosomal and plasmid-mediated genes, including *bla_TEM_*, *bla_SHV_*, and *OXA-1* [18]. Briefly, 2 μL DNA aliquot was subjected to each multiplex PCR in 20 μL reaction mixture containing 10.6 μL of nuclease-free water, 3 μL of the template, 4 μL of ready 5× Hot FIREPol® Blend Master Mix Ready to load (Hot FIREPol® DNA Polymerase, Proofreading enzyme, 5× Blend Master Mix Buffer, 12.5 mM MgCl2, 2 mM dNTPs), and a variable concentration of specific-group primers (Table-1). Amplifications were carried out as follows: Initial denaturation at 94°C for 10 min followed by 30 cycles of 94°C for 40 s, 60°C for 40 s and 72°C for 1 min, and a final elongation step at 72°C for 7 min. Amplified samples were evaluated after running at 100 V for 1 h on a 2% agarose gel containing ethidium bromide (0.5 μg/mL). A 100 bp DNA ladder (BioLabs, USA) was used as a size marker.

### Pulse-field gel electrophoresis (PFGE)

A total of 41 EHEC isolates were used in the PFGE using restriction enzyme digestion with XbaI enzyme (Thermo Fisher, USA) according to previously published protocols [19]. Gel electrophoresis was performed using the Chef Mapper XA PFGE system (Bio-Rad) under the following conditions: Initial switch time: 2.2 s; final switch time: 54.2 s; run time: 18 h; angle: 120°; gradient: 6.0 V/cm; temperature: 14°C; and ramping factor: linear. The electrophoresis gels were stained using ethidium bromide and visualized under ultraviolet light. The genotypic relatedness was determined using PFGE DNA fingerprint subtypes. The analysis of the bands was carried out using the Dice coefficient and the unweighted pair group method with arithmetic averages clustering methods with an optimization and position tolerance of 1.0%. Analysis of PFGE gel patterns was performed using BioNumerics software version 3.5 (Applied Maths, Belgium).

## Results

A total of 194 (47.3%) *E. coli* were isolated from 100 (44%) human, 100 (46%) cattle, 35 (74%) sheep, 35 (54%) goats, and 100 (39%) broilers fecal samples and 40 (50%) ground beef meat samples (Table-2).

**Table-2:** Serotypes and virulence genes in enterohemorrhagic *Escherichia coli* isolated from fecal samples of humans, animals, poultry litter, and ground beef meat.

Sample source (n)	Number of isolates (%)	H7	O157	O157:H7	O157:H	Stx1	Stx2	Non-O157
Human (100)	44 (44)	8 (18.2)	1 (2.3)	0 (0)	1 (2.3)	0 (0)	0 (0)	8 (18.2)
Cattle (100)	46 (46)	15 (32.6)	7 (15)	4 (9)	3 (6)	0 (0)	0 (0)	1 (2)
Broilers (100)	39 (39)	17 (43.6)	6 (15)	2 (5)	4 (10)	0 (0)	0 (0)	6 (15)
Sheep (35)	26 (74.3)	8 (30.7)	6 (31)	3 (11)	3 (11)	2 (8)	0 (0)	6 (23)
Goats (35)	19 (54.3)	4 (21)	8 (21)	4 (21)	4 (21)	1 (5)	0 (0)	1 (5)
Beef meat (40)	20 (50)	13 (65)	7 (35)	6 (30)	1 (5)	0 (0)	1 (5)	0 (0)
Total (410)	194 (47.3)	65 (33.5)	35 (18)	19 (10)	16 (8)	3 (1.5)	1 (0.5)	22 (11)

### Serotypes and virulence factors

Serotypes and virulence genes in EHEC isolated from human, animals’ fecal samples, poultry litter, and ground beef samples are presented in Table-2. Fifty-seven (29.4%) isolates out of 194 were identified as EHEC of which 19 isolates were O157:H7 and 16 were O157:H serotypes. Twenty-two isolates (11%) were non-O157.

A total of 22 (38.5%) out of 57 isolates belonged to five serotypes (O26, O111, O113, O103, and O8). In human samples, O111, O26, and O113 serogroups were detected in a total of 8 (18.6%) out of 43 isolates. In cattle samples, only 1 isolate (2.5%) was found to be O8. In sheep samples, the O111, O26, and O103 serogroups were detected in a total of 6 (33.3%) out of 18 isolates. In goat samples, only 1 isolate (9%) was detected and belonged to O26 serogroup. In poultry litter samples, 6 (18%) isolates were determined as O8 serogroup. In ground beef meat samples, none of the EHEC serogroups was detected.

Thirty-five isolates belonged to *E. coli* O157 serogroup, 19 of them were O157:H7. However, O157:H7 was not detected in any of the samples obtained from animals or ground beef meat. In human samples, one O157 serotype was detected. None of the 44 human *E. coli* isolates carried *sxt1* or *sxt2* genes. In cattle samples, seven isolates were found to be O157 serotype, none of which carried the *stx1* or *stx2* genes. In poultry litter, six isolates were found to be O157 serotype, with no *stx1* and *stx2* genes. In sheep samples, six isolates were found to be O157 serotype, two of which carried the *stx1* gene. In goat samples, eight isolates were found to be O157 serotype, one of which carried the *stx1* gene. In ground beef meat samples, seven isolates were found to be O157 serotype, one of which carried the *stx2* gene.

### Antimicrobial sensitivity patterns

EHEC isolates showed high phenotypic resistance patterns against AX, CN, CL, and doxycycline. Thirty-two (56%) of the EHEC isolates showed 100% resistance to at least two beta-lactams antibiotics, 24 (75%) of which were determined as ESBL producers. Only 14 isolates of the ESBL producers expressed the *bla_SHV_* gene, 19 isolates expressed the *bla_TEM_* gene, 4 expressed both genes, and none carried the *OXA-1* gene.

### PFGE

**Figure-1:**
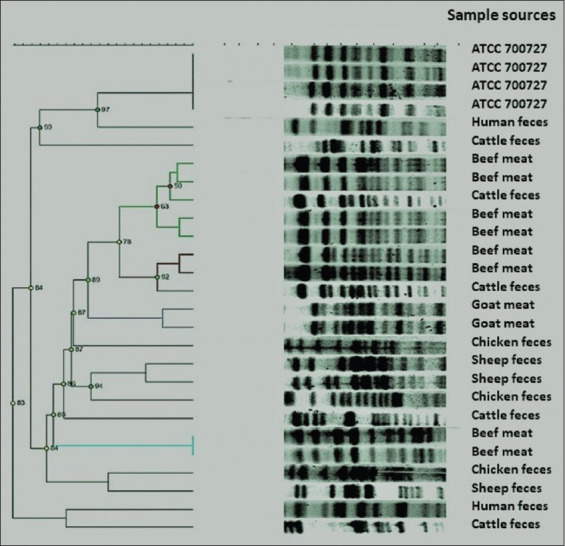
Dendrogram image showing the relatedness of pulsed-field gel electrophoresis patterns for *Escherichia coli* isolates from fecal samples of human and animals, poultry litter, and ground beef samples.

PFGE analysis revealed two clusters containing isolates from cattle fecal samples and ground beef meat (Figure-1). The isolates within these clusters are closely related and can be considered identical. Each of the other clusters in the dendrogram contains only two isolates. There are two clusters of goat fecal samples, one cluster of sheep fecal samples and one cluster of ground beef meat samples that did not show any correlation with other ground beef meat cluster and cattle fecal samples cluster. The isolates from human fecal samples are not related to any other tested isolates.

## Discussion

The present study reports the molecular characteristics of EHEC isolates from human and animal sources in Jordan. In this study, a total of 194 *E. coli* isolates were cultured from human (44%), cattle (46%), sheep (74%), goats (54%) fecal samples, poultry litter (39%), and ground beef meat (50%) samples. These results are similar to previously reported findings in various parts of the world [20,21]. High percentages (29%) of *E. coli* isolates in this study were classified as EHEC in various sample sources (20% of human samples, 17% of cattle samples, 46% of sheep samples, 47% of goat samples, 31% of poultry samples, and 35% of ground beef meat samples). These findings are higher than the results reported in Iran and the USA, in which EHEC were only recovered from 7.6% and 9% of broiler chickens’ samples and cattle fecal samples, respectively [22,23].

In the present study, the frequency of *E. coli O157* serotypes varied according to the sample source. The incidence of *E. coli O157* was highest in sheep samples (30.7%) and lowest in human samples (2.3%). Similarly, the incidence of *O157* in Germany, Ghana, Iran, and Iraq among sheep, cattle, ground beef meat, and goats was 21%, 12.7%, 8.2%, and 25%, respectively [21-26].

The occurrence of *stx1* in the current study was different from the Norwegian study which showed that *sxt1* of EHEC non-O157 strain (O26) was detected in sheep fecal samples at a rate of 17.9% [27]. In this study, all isolates that carried the *stx1* gene were O26, and this finding is similar to that found in Australia [28]. On the other hand, the *stx2* gene was detected in only one EHEC isolate (5%) from beef ground meat. These results are different from previously reported data that showed 28% of 14 EHEC isolates carried *stx2* [24]. In another study, 61% of non-O157 EHEC isolated from sheep and goats carried the *stx1* gene, 21% carried the *stx2* gene, and 18% carried both genes [29].

PFGE analysis of *E. coli* isolates from human, animal, and ground beef meat samples in this study revealed different clusters. These results showed high similarity (92%) among isolates from ground beef meat and those from cattle fecal samples, which suggest a cross-contamination during processing. In addition, the isolates from beef meat were classified as O157:H7 serotype and those of cattle fecal samples were O157:NM. These results suggest a close genetic relationship between the isolates of EHEC O157 from ground beef meat and cattle origin. However, the results showed no relatedness between isolates from animal origin and human origin. These results are in agreement with the previously reported findings, which suggest no transmission of *E. coli* between animals and humans [30].

## Conclusion

The results of this study indicate widespread ESBL EHEC among humans, animals, and ground beef meat samples. These results represent an important alarm that requires the implementation of appropriate preventative measures by both human and animal health sectors to prevent cross-transmission of this important foodborne pathogen.

## Authors’ Contributions

YHT and SNE: Conceived, designed, and supervised the study. ZBI: Analyzed the data and edited the final manuscript. AAA: Collected the samples, performed laboratory analysis, and wrote the manuscript. All authors read and approved the final manuscript.
